# PASS Theory of Intelligence and Its Measurement Using the Cognitive Assessment System, 2nd Edition

**DOI:** 10.3390/jintelligence12080077

**Published:** 2024-08-06

**Authors:** Jack A. Naglieri, Tulio M. Otero

**Affiliations:** 1 Department of Psychology, George Mason University, Fairfax, VA 22030, USA; 2 Department of Psychology, California Southern University, Chandler, AZ 85286, USA; braindoctmo@gmail.com

**Keywords:** intelligence, PASS theory of intelligence, PASS neurocognitive theory, cognitive assessment system, second edition, CAS2, equitable assessment of intelligence, pattern of strengths and weaknesses, SLD, ADHD, ASD, test bias, test fairness, discrepancy consistency method

## Abstract

The goal of this paper was to describe the context within which the PASS theory of intelligence was conceived and the reasons why this theory was used to guide the construction of the Cognitive Assessment System and the several versions of the Cognitive Assessment System, 2nd Edition. We also discuss validity issues such as equitable assessment of intelligence, using PASS scores to examine a pattern of strengths and weaknesses related to academic variability and diagnosis, and the utility of PASS scores for intervention. We provide summaries of the research that informs our suggestions that intelligence testing should be theory-based, not constrained by the seminal work of test developers in the early 1900s, and neurocognitive processes should be measured based on brain function.

## 1. Introduction

During most of the 1900s and a quarter of the 2000s, group and individually administered intelligence tests played a key role in education and psychology. Since Terman (1916) built upon the work of Binet and Simon to create the 1916 Stanford–Binet, the scores his test provided have changed the course of countless peoples’ lives—for some in good and others in bad ways (i.e., eugenics movement). Intelligence tests have played an important part in a comprehensive assessment, as recognized by Bronner et al. (1927), who wrote, “Investigation of the mental capacities of human beings may rationally be considered a matter of prime importance for the individual and for civilization” (p. v). Today, intelligence tests are one of the most widely used tools by psychologists, and the scores these tests yield are the foundation of important decisions about children and adults (Benson et al. 2019).

Despite the widespread use and the enormous impact intelligence tests have had, there has been and continues to be considerable controversy over their value, test fairness, interpretation, and even how to define and measure intelligence (e.g., APA 2021; Ackerman 2022; Kaufman et al. 2016; Naglieri and Otero 2017). Of all the issues surrounding intelligence tests, perhaps the two most important questions are: What kind of questions should be used to measure intelligence? And do these questions measure constructs that are defined by a *theory* of intelligence? 

With reference to the Binet–Simon Scale, Binet and Simon (1916) stated that: “The scale… is not a theoretical work; it is the result of long investigations…in the primary schools of Paris, with both normal and sub normal children” (p. 41). They criticized “authors who have made a specialty of organizing new tests according to theoretical views, but who have made no effort patiently try them out in the schools” (p. 41). Binet and Simon’s examination of their tests helped them identify children in need of special education because of intellectual disability, and they made a distinction between the measure of intelligence and their method of classifying degrees of subnormal intelligence (Binet and Simon 1916, p. 40, footnote 1). The tests they created were designed to be “simple, rapid, convenient, precise, heterogeneous, holding the subject in continued contact with the experimenter, and bearing principally upon the faculty of judgement” (p. 41). They did notice that “there are tests which require knowledge outside the intelligence of the child… that he has learned… from his parents or friends…and there are tests too exclusively scholastic, we have thought well to suppress” (Binet and Simon 1916, p. 275). They continued, “This verbal superiority must certainly come from family life; the children of the rich are in a superior environment from the point of view of language; they hear a more correct language and one that is more expressive” (p. 320). In contrast, Terman *included* items dependent upon school learning in the 1916 version of the Stanford–Binet. He believed that “intelligence at the verbal and abstract levels is the highest form, the *sine qua non*, of mental ability” (Freeman 1955, p. 127). Subsequently, the Stanford–Binet was criticized because it relied “much too heavily with verbal and abstract material, thus penalizing the individual who for whatever reason, had been handicapped… by lack of opportunity to acquire and develop the use of the English language” (Freeman 1955, p. 127). Terman’s perspective on intelligence testing influenced the content of the US Army Alpha and Beta Tests (Yoakum and Yerkes 1920; Yerkes 1921) via his student Arthur Otis.

Arthur Otis was instrumental in the development of the Army Mental Tests, which, in turn, influenced David Wechsler, who was trained in the School of Military Psychology (Yerkes 1921) and aware of the Army Mental Tests as well as the Binet–Simon and other scales. The intelligence test Wechsler ultimately published in 1939 included subtests that were similar to those found in the Army Alpha and Beta (Wechsler 1941; Matarazzo 1972). Decades later, McNemar (1964) noted that despite different titles and authors, intelligence tests developed to that point were “little more than tests of general intelligence, and thus are direct descendants of the Alpha and Beta which, in turn, were descendants of the Binet-Simon” (Wechsler 1941; Matarazzo 1972, p. 46).

Pintner and Paterson (1925) noted that “Theoretical considerations have lagged behind the practical application of mental tests. We have been measuring intelligence long before we have decided as to what intelligence really is” (p. 1). The notion of general ability enunciated by Stern (1914) was widely accepted, yet as Pintner (1923) explained, “We did not start with a clear definition of general intelligence… psychologists borrowed from every-day life a vague term implying all-round ability… and has been and still is attempting to define it more sharply and endow it with a stricter scientific connotation” (p. 53). Pintner foresaw the considerable efforts psychologists would make over the next 100 years to better understand and interpret what could be described as traditional intelligence tests. Perhaps an evolutionary change is needed.

It seems reasonable that the first question that should confront any intelligence test developer has been and should be, “What theory of intelligence will the test be built upon?” The question of test content would then be guided by the theory of intelligence. It also seems reasonable that the theory of intelligence should be based on an understanding of neurocognitive brain functions. We will provide a description of how neurocognitive abilities have been used to define intelligence and how test questions could be devised to represent those abilities. We will align those neurocognitive abilities to recent research on neural networks and examine various aspects of the validity of one such approach.

## 2. The PASS Neurocognitive Theory

The PASS theory (Naglieri and Das 1997b) is rooted in the conceptualization of brain function as described by Alexander Luria (1966, 1973b, 1980). Das and Naglieri utilized Luria’s description of the basic neurocognitive processes to define intelligence (Das et al. 1994; Naglieri and Das 1997b; Naglieri and Otero 2017). Luria hypothesized that human cognitive functions can be conceptualized within a framework of three separate but interrelated brain systems that provide four basic psychological processes. These brain systems are referred to as functional units because the neurocognitive mechanisms associated with each work in separate but interrelated components, namely, Planning, Attention, Simultaneous, and Successive, of basic psychological processes. Each of these neurocognitive abilities will be described in the sections that follow.

### 2.1. Planning

Planning is a neurocognitive ability used when a person decides how to complete a task using a strategy, self-monitoring, and self-correction, especially in novel situations (Goldberg 2009). Planning provides for the generation of new ways to solve problems, especially in situations where no method or solution is immediately apparent. Planning ability is also used when individuals reflect on events following a task that was completed, recognizing what worked and what did not work and anticipating other viable options to consider in the future. 

CAS2 and CAS2: Brief subtests on the Planning scale vary in their content, but they all present the examinee with novel problems to solve. The examinee who creates a strategy completes the task more efficiently and, therefore, obtains a higher score. The Planned Codes subtest is a good example of a task that can be solved using a strategy. The subtest requires the child to write a specific letter code under the corresponding letter (e.g., XO for A, OX for B, etc.). Children often use a strategy such as completing all the As and then the Bs, which results in higher scores than those who do not (Naglieri et al. 2014b). All three subtests on the Planning scale are more efficiently completed using a strategy.

Teacher observations of a student’s classroom behavior included on the CAS2: Rating Scale (Naglieri et al. 2014d) can provide insight into a student’s ability to use planning. For example, the teacher’s observations about how well the student solves novel tasks and how well a student can think of several ways to solve a problem can illustrate if and how a student is using Planning. It is important to consider, however, if the classroom instruction is very structured and each student is taught to use the same method of solving problems, then the behavior in the class will reflect how well the student is following directions rather than how well the student *could* develop a variety of solutions.

### 2.2. Attention

Attention is a neurocognitive ability used to selectively focus on a specific stimulus while inhibiting responses to other stimuli. Attention is an essential component of intelligent behavior because it provides cortical arousal and higher forms of attention and is required for the recruitment of other neurocognitive processes. Optimal conditions of arousal are needed for the more complex forms of attention involving “selective recognition of a particular stimulus and inhibition of responses to irrelevant stimuli” (Luria 1973a, p. 271). Higher forms of attention include focused and selective cognitive activity, shifting attention based on salience, and resistance to distraction. The longer attention is needed, the more the activity requires effort.

As an example, the Expressive Attention subtest in the CAS2 requires the student to identify one aspect of a target stimulus (e.g., the color blue) and resist responding to distractions (e.g., the red word written in blue ink) as in the Stroop test (Stroop 1935). This task requires resistance to distraction and focused, selective, sustained, and effortful activity (Lezak 1995). Focused attention allows for the identification of a specific stimulus, selective attention provides the inhibition of responses to distracting stimuli, and sustained attention provides continued effort over time.

Classroom behaviors observed by the teacher on the CAS2: Rating Scale can reflect a student’s ability to attend and resist distractions over time. For example, behavioral evidence of good attention can be noted when the teacher observes how well a student can stay focused on their work despite distractions in the classroom as well as distracting thoughts. Similarly, solving a math problem such as 2 + 6 − 1 =? involves careful attention to the numbers and the signs in addition to knowing the math facts. 

### 2.3. Simultaneous

Simultaneous processing is a neurocognitive ability used to integrate separate stimuli into a single whole or interrelated group. This ability is used when separate elements must be combined into a conceptual whole. This may involve visual–spatial as well as linguistic stimuli that require comprehensive grammatical structures. The spatial aspect of Simultaneous ability involves the perception of stimuli and their interrelationships as a whole and the use of visual images. The grammatical dimension of Simultaneous processing provides a way to integrate words into ideas through the comprehension of word relationships, prepositions, and inflections so the person can obtain meaning. It is important to recognize that Simultaneous processes can involve nonverbal as well as verbal content. 

The Verbal Spatial Relations subtest is a good example of a task that demands Simultaneous processing. The test requires that the examinee understand the interrelationships of objects presented in six different scenes. The task is to identify which scene corresponds to a verbal statement (e.g., “which picture shows a ball under the table?”) provided by the examiner. The other two subtests on the Simultaneous scale required understanding relationships, for example, among shapes (i.e., Matrices and Figure Memory).

Classroom behaviors can reflect the use of Simultaneous processing as measured by the CAS2: Rating Scale. Students who prefer hands-on materials and visual–spatial tasks like drawing designs, especially three-dimensional ones, and those who are good at patterns and complex shapes are usually good at Simultaneous processing. Simultaneous neurocognitive ability is also essential for identifying words as a whole (e.g., sight words), understanding grammar (Luria 1982) and patterns in the spelling of words, verbal concepts, and reading comprehension dependent on getting the big picture. 

### 2.4. Successive

Successive processing is a neurocognitive ability used when information is arranged in a specific sequence in which each part follows the other in a strictly defined order. Successive processing is used to manage any activity that is arranged in a sequence, for example, the formation of sounds and movements into a specific order. This ability is necessary for recalling information in order and understanding a statement based on the syntax of the language, as well as phonological analysis (Luria 1982; Lezak 1995). Successive processing is important for the initial acquisition of reading, decoding, remembering the sequence of motor movements, speech articulation, listening comprehension, and other tasks that require following sequential order. 

The CAS2 and CAS2: Brief subtests that are used to measure Successive processing vary in their content, but all assess how well a student can manage a sequence of stimuli. The tasks demand repeating a sentence using the correct series of words as well as comprehension of sentences that are understood only by appreciating the sequence of words. The CAS2 also measures successive processing across auditory and visual modalities using the Word Recall and Visual Digit Span tests, respectively. 

Classroom behaviors and those included in the CAS2: Rating Scale can reflect a student’s facility working with information in order. For example, following a series of directions given by the teacher demands Successive processing. Similarly, the examination of the sequence of a series of events is also involved in reading, especially initial reading, to decode unfamiliar words and spelling. Successive processing is critical when a student is presented with confusing words and must focus carefully on the pronunciation of sounds in order. 

## 3. Measurement of PASS Theory Using CAS2

### 3.1. CAS2, CAS2: Brief & CAS2: Rating Scale

**Figure 1:**
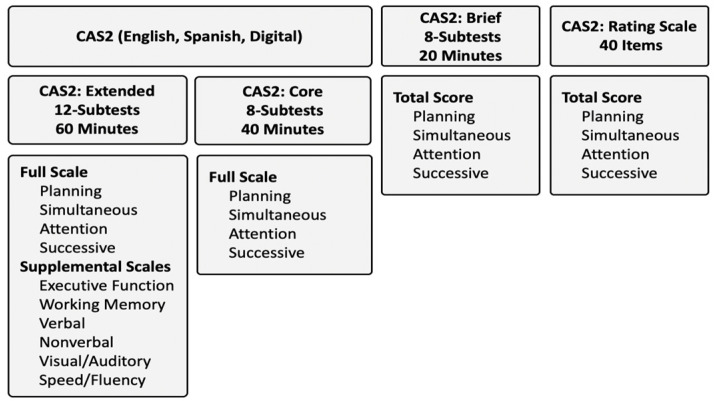
Cognitive Assessment System—2nd Edition.

There are several ways to measure PASS neurocognitive abilities using the Cognitive Assessment System, Second Edition (CAS2; Naglieri et al. 2014b). Practitioners have the option to use the CAS2 12-subtest Extended version, which yields standard scores for all subtests, the Full Scale, Planning, Attention, Simultaneous, Successive scales, and six Supplemental scores (see Figure 1). The CAS2 8-subtest version yields all subtests and five scores for the PASS and Full Scale. Both versions, as well as the English, Spanish (Naglieri et al. 2017), and Digital (Naglieri and Otero forthcoming) formats, are scored using *CAS2: Online Scoring and Report System* (Naglieri et al. 2014a), which generates all scores and an interpretive report. The CAS2: Brief comprises four subtests that yield five standard scores (PASS and a Total Score). The eight subtests on the CAS2 Core are the same as those on the CAS2 Extended, but the subtests on the CAS2 Brief are similar but not the same as those on the CAS2. Finally, the CAS2: Rating Scale comprises 10 items per PASS scale completed by a teacher and yields PASS and Total scores. Standard scores set at a mean of 100 and a standard deviation of 15 are provided. 

One unique feature of the CAS2 and CAS2 Brief is that once the standard administration directions are provided to the examinee, the examiner is allowed to use alternative means to ensure the examinee clearly understands what is required to complete the task. If the child does not seem ready or appears in any way confused or uncertain, the examiner is allowed to provide a brief explanation if necessary. This is intended to give the examiner the freedom to explain what the child must do in whatever way necessary to ensure that the child understands the task, including gestures, verbal statements, or communication in any language. The intent is to give the examiner full decision-making to explain the demands of the subtest and to ensure that the child understands what to do. To date, we are not aware of any other measure of cognitive ability allowing this additional method. For more information about the different versions of the CAS2, including, for example, PASS scale variability, psychometric analysis of differential item functioning, and score interpretations, see the respective Manuals. 

The development and cultural adaptation of the CAS2 Español began with an initial translation of the CAS into Spanish, undertaken in 2000 by a group at the University of Puerto Rico led by Wanda Rodriquez. These researchers used a method called “back translation”, in which the test is translated from English to Spanish and then it is translated back from Spanish to English. The administration and scoring manual, the test’s written materials, and the test scoring sheet were translated using this method. The twelve CAS subtests were divided into two equal groups, and each group was assigned to a pair of translators. Each translator on the team worked independently on six subtests, and once the subtests were translated, the two translators on the same team compared their translations. Any disagreements were discussed, and when necessary, teams consulted a translator on the other team. When agreement was reached on the translation of their six subtests, one translator from each team joined to determine the consistency of the vocabulary used in the whole test. Once these processes were completed, the product was presented to two psychologists with broad experience in instrument translation, and they, in turn, checked for coherence between the English and Spanish versions. A similar approach was used for the CAS2—Spanish, but with a larger group of experts that included psychologists and educators from different geographical locations with knowledge of different Spanish dialects.

Within the normative sample of the CAS2, Hispanic males and females were proportionally well represented, consistent with the 2011 US census. According to the US census, Hispanics ages 5 to 21 years constituted 21% of the population, and the CAS2 matched this within its normative sample. The validity of using PASS and CAS with Hispanics has been achieved through several means. Several studies have examined the CAS scores for racial and ethnic group differences. Naglieri et al. (2007) found that CAS Full Scale scores for Hispanic and White children differed by 4.8 points when demographic differences were statistically controlled. They also reported that the correlations between CAS scores with achievement did not differ significantly for the Hispanic and White samples. Naglieri et al. (2007) compared PASS scores obtained on the CAS when administered in English and Spanish to bilingual children referred for reading problems. The children earned similar Full-Scale scores on the English (mean of 84.6) and Spanish (mean of 87.6) versions of the CAS, and the scores from the two versions were highly correlated (r = 0.96). Additionally, Otero et al. (2013) studied the performance of referred Hispanic ELLs on the English and Spanish versions of the CAS and reported that the Full-Scale scores on the English (mean of 86.4) and Spanish (mean of 87.1) versions were very similar and highly correlated (r = 0.99, corrected for range restriction). These findings for the CAS suggest that ability may be more fairly assessed across race and ethnic groups with the PASS neurocognitive approach.

### 3.2. CAS2 Versions

The rationale behind the development of the various versions of the CAS2 was driven by the ways different practitioners could obtain and use PASS scores. For example, the CAS2 8- and 12-subtest versions offer a comprehensive examination of a person’s neurocognitive abilities, which can be used for diagnostic decision-making and instructional planning. The CAS2: Brief can be used as a screening tool for possible learning problems and decisions related to instructional planning, as well as for re-evaluations and gifted identification. The CAS2: Rating Scale can be used with the CAS2 and CAS2: Brief to determine the similarity of the scores across these measures. For a full discussion of these versions of the CAS2, see Essentials of CAS2 Assessment (Naglieri and Otero 2017) and the respective test Manuals.

### 3.3. Neuropsychological Underpinnings

The CAS and the CAS2 were created using the PASS Theory as a guide; the theory itself was based on Luria’s understanding of how the brain operates. Although sophisticated neuroscientific resources that exist today did not exist in the times of Luria, his understanding of how the brain works still stands as valid (e.g., Zelazo and Carlson 2020). The functional units of the brain he described can today be understood as functional networks. These networks involve several cortical and subcortical structures that are in constant flux of neural activity based on environmental demands. For example, studies using functional imaging technology (Avram et al. 2013; Yeo et al. 2011; Zaytseva et al. 2014) have shown that each area of the brain participates in numerous large and small-scale functional systems within and across cortical and subcortical brain structures. Supportive research in neuroscience literature has shown that functional systems combine and dissolve at different times and on fast timescales across tasks (Sporns et al. 2021; Koziol et al. 2014, 2016). These networks have a profound impact on constructs such as attention, executive function, learning and memory, and information processing. Luria (1973b) clearly stated that cognitive activity is the result of an interplay of complex functional systems, yet each system makes unique contributions. His assertion remains true today.

No part of the brain functions in isolation, and any given cortical region has a degree of information-processing specificity for a cognitive ability or part of cognitive operations (Friston 2002; Johnson 2005; Passingham and Rowe 2015; Passingham 2021). This specificity is referred to as functional specialization. As originally put forth by the work of Luria, effective performance on any given task is characterized by the functional integration of distal brain regions. This union represents the transitory, dynamic, context-specific communications that transfer information via subsets of anatomical connections among a limited number of brain regions engaged by a cognitive process (Koziol and Stevens 2012). 

Luria’s work on developing cognitive constructs and corresponding behaviors as manifestations of the operations of brain systems became known as functional units of the brain. In more recent terminology, this is equivalent to the well-recognized concept of brain networks. Table 1 represents a conceptualization of how PASS processes relate to functional units and how these relate to large-scale neural networks. For a detailed discussion on PASS processes, functional units, and their relationship to neural networks, see Naglieri and Otero (2011).

The basic neurocognitive processes (PASS) responsible for the cognitive activity underlying intelligence and behavior represent a “working constellation” (Luria 1966, p. 70) of networks. Just as a variety of neural networks operate in a dynamic manner for a particular task, a person may execute the same task using any combination of the PASS processes, along with the application of the person’s knowledge and skills. Although completing most any task is accomplished through the integration of all processes, not every process is equally involved in every task. In addition, a task may be approached using varying combinations of processes, depending on how the task was initially taught or learned. For example, tasks like math calculation may be dominated by a single process (e.g., planning), while tasks such as reading decoding may be strongly related to another process (e.g., successive) while also recruiting other neurocognitive processes. Reading comprehension of familiar text may, for example, recruit both Simultaneous and Successive processes, while reading something composed of unfamiliar content may require an additional process to be recruited. The dynamic way PASS abilities intersect provides a way of using the neurocognitive processing strengths to address the PASS weaknesses involved in the learning process. 

### 3.4. Test Content and Equitable Assessment 

Now that the PASS theory and its operationalization in the CAS2 have been presented, we can begin to cover the practical implications of having a theoretical basis for a test of intelligence. Recall that two interrelated issues raised at the start of this paper are closely related to the equitable assessment of intelligence: (a) the need for a *theory* of intelligence and (b) test content should be aligned with the theory of intelligence. A theory of intelligence should provide the vision for the cognitive structure of the tasks used to measure intelligence from a theoretical perspective. For example, the PASS theory provided a description of what kind of thinking the subtests should evoke. From our theory, this means the following:

- Planning subtests should measure how well a person creates and uses strategies to complete a task.
- Attention subtests should measure how well a person can focus and resist distractions.
- Simultaneous subtests should measure how well a person can understand relationships among things.
- Successive subtests should measure how well a person can manage the sequence of a task.

When the Cognitive Assessment System was initially built, the measurement of the PASS basic psychological processes could have been achieved using tasks that demand knowledge. For example, written composition was used as a measure of Planning by Das et al. (1979a) but a subtest like that would have reflected knowledge as well as Planning and, therefore, it was not deemed appropriate. This issue is important because the concept of fairness is described in the Standards for Educational and Psychological Testing (AERA et al. 2014), which has two components—psychometric test bias and test content. Psychometric test bias has been examined. For example, Naglieri et al. (2005) reported similar correlations between PASS scores on the CAS and academic achievement test scores across race. Naglieri et al. (2007) found similar correlations with achievement for Hispanic and White students. Naglieri et al. (2014c) reported that none of the dichotomously scored items on the CAS2 were found to be biased across gender, race, and ethnicity using differential item functioning (DIF) analysis. Factorial invariance of PASS scores was reported by Naglieri et al. (2013). They reported multigroup confirmatory factor analysis results, which supported the configural invariance of the CAS factor structure (i.e., the PASS scales) between Italian (N = 809) and American children in 5- to 7-year-old and 8- to 18-year-old groups. These analyses are informative, but in addition to psychometric test bias, test content is also related to test fairness. 

The Standards for Educational and Psychological Testing state that “opportunity to learn…can influence the fair and valid interpretations of test scores (p. 56)”. “Opportunity to learn is a fairness issue when [there is] differential access to opportunity to learn for some individuals and then holds those individuals who have not been provided that opportunity accountable for their test performance… [even if the test] may not be biased” (p. 57). Equitable assessment can be maximized when all examinees have an equal opportunity to display their ability to answer the questions on a test, and fairness can be thwarted by the inclusion of questions that demand knowledge some may not have had the opportunity to acquire. The standards also state that “Test users should be alert to potential misinterpretations of test scores… [and] take steps to minimize or avoid foreseeable misinterpretations and inappropriate uses of test scores” (p. 143). However, there is a history of using tests that demand knowledge (e.g., vocabulary, word analogies, arithmetic word problems) to measure intelligence, and in some instances, very similar test questions appear on intelligence and achievement tests (Naglieri 1999; Schneider 2013). 

The similarity in content across intelligence and achievement tests was noted by Schneider (2013) when he wrote, “inspection of the contents of most IQ tests reveals that many test items could be repurposed as items in an achievement test (e.g., vocabulary, general knowledge, and mental arithmetic items) (p. 287)”. Fagan and Holland (2006) suggested that differences in knowledge between African Americans and Whites were related to differences in intelligence test scores, which could be eliminated when there is equal opportunity for exposure to the information to be tested. Other researchers (e.g., Goldstein et al. 2023) have suggested that intelligence tests that do not rely on knowledge would be more equitable. Despite these cautions, intelligence tests continue to include items that demand verbal knowledge and general information and use arithmetic word problems (Brulles et al. 2022). The continued use of questions that demand knowledge in a *cognitive* test has historical precedence but warrants justification.

Terman defended the use of verbal tests in the 1916 Stanford–Binet because he believed responses to verbal questions represented the highest form of mental ability. More recently, Lohman et al. (2008) argued that “verbal and quantitative abilities add importantly to the prediction of academic success” (p. 276). Some might suggest the logic behind this position to be considered circular. That is, verbal and arithmetic questions are good measures of intelligence because they correlate with verbal and math achievement test scores. Similarly, Lubinski and Benbow (2021) wrote, “the SAT-Mathematics and SAT-Verbal composite is an excellent measure of IQ or general intelligence” (p. 4). Lynn (2010) asserted that “Scores on [reading comprehension and mathematics can be] used as a proxy for IQ [because a] reading test is a measure of verbal comprehension and [a] mathematics test is a measure of “quantitative reasoning”, and both of these are major components of general intelligence (e.g., Carroll 1993, p. 597; McGrew and Flanagan 1998, pp. 14–15) (p. 95).” 

Lynn (2010) used the 2007 PISA reading and math scores as a measure of intelligence (IQ) to compare children across regions in Italy. He concluded, “The lower IQ in southern Italy may be attributable to genetic admixture with populations from the Near East and North Africa” (p. 9). Lynn’s conclusion was challenged by D’Amico et al. (2012), who found little differences between southern and northern Italian children on Raven’s Progressive Matrices (Raven 1954) and PASS scores from the Italian version of the Cognitive Assessment System (Naglieri and Das 2006). D’Amico et al. argued that the differences in the PISA verbal and math scores reflected differences in children’s educational opportunities, not intelligence, and their results suggested that measuring intelligence with tests that are not dependent upon knowledge was more valid and equitable. Regardless of the rationale for the use of intelligence tests that demand knowledge, test content has considerable implications for fair assessment.

The correspondence between test questions that demand knowledge and test fairness across intelligence tests can be understood by the examination of average test score differences across racial and ethnic groups. Brulles et al. (2022) explored this question for group and individually administered intelligence tests and found larger race and ethnic differences on tests that include knowledge than tests with minimal knowledge. Table 2 provides a larger summary of the available research. The results suggest that those tests that require knowledge yield large score differences in total standard scores by race (average difference by race of 9.4 standard score points) and ethnicity (Mn = 6.6). In contrast, tests that require minimal knowledge yield smaller average score differences by race (Mn = 4.3) and ethnicity (Mn = 2.9). These findings suggest a relationship between intelligence test content and test equity. 

Perhaps the best test of the hypothesis that knowledge leads to equity problems for group-administered IQ tests such as the CogAT and OLSAT, which provide verbal, nonverbal, and quantitative scores, is addressed with the results presented for the Naglieri General Ability Tests: Verbal, Nonverbal and Quantitative (Naglieri et al. 2021; Selvamenan et al. 2024). Race, ethnicity, gender, and parental education level differences on the Verbal, Nonverbal, and Quantitative tests of the Naglieri General Ability Tests were examined. These tests were explicitly designed to measure general ability without the knowledge demands found in traditional intelligence tests. That is, they have features that the authors suggested make them appropriate for diverse populations of students, which include the following: (a) each test’s directions were delivered using an animated scene like that experienced by the student being tested so no verbal instructions are used; (b) no verbal response is required of the student; (c) the verbal test requires the student to identify a verbal concept represented in pictures and determine which image does not represent the concept; (d) the quantitative test uses questions that require close examination of the relationships among numbers and/or symbols, numerical sequences, and patterns involving only basic math; (e) the nonverbal test uses questions that require examination of shapes presented in a pattern, sequence, spatial orientation, and other distinguishing characteristics to arrive at the correct answer in a manner similar to the Naglieri Nonverbal Ability Test, 3rd Edition (Naglieri 2016). These three tests have different content, but factor analytic results provide support for their validity as measures of a broad general ability factor (Naglieri et al. 2021). The results for these three tests, presented in Table 2, support the view that the academic knowledge required in traditional intelligence tests likely contributes to differences across race and ethnicity. 

It is important to recall that many psychologists have cautioned against including questions that demand knowledge in intelligence tests. These voices were largely ignored, and the early development of intelligence tests has had a lasting impact on the content of intelligence tests used today. We suggest that a fair assessment of intelligence must be achieved, and this is more likely to occur if a neurocognitive approach to test development and test content is followed. Perhaps intelligence tests should be conceived and developed on a theory of intelligence, and the test’s questions should measure the kind of thinking and problem-solving that is defined by the theory. To ensure that all students have an equal opportunity to do as well as they can on a measure of intelligence, test questions should measure *how well students can answer the questions by thinking in a way that is not confounded by how much they know*. This is the approach that was used when the Cognitive Assessment System was initially created in 1984.

## 4. Empirical Support for the PASS Theory as Measured Using the CAS2

The initial effort that led to the PASS theory was initiated by Das and colleagues (Das et al. 1975, 1979b, 1994) and included an extensive analysis of the methods used by Luria and related measures used in neuropsychology, as well as cognitive and educational psychology. The possible methods that could be used to measure Luria’s conceptualization of basic psychological processes and ultimate operationalization using the CAS were summarized in several books (e.g., Das et al. 1994; Kirby 1984; Kirby and Williams 1991; Naglieri 1999; Naglieri and Das 1997a; Naglieri et al. 2014b; Naglieri and Otero 2011, 2017). The publication of the CAS2 (Naglieri et al. 2014b) and the CAS2: Brief (Naglieri et al. 2014c) test Manuals provided additional evidence for PASS theory and were further described in Essentials of CAS2 Assessment (Naglieri and Otero 2017). We summarize additional validity research in the sections that follow.

### 4.1. PASS Correlations with Achievement

Psychologists often rely on the examination of intelligence test scores to understand academic strengths and weaknesses and to anticipate future academic achievement. This makes understanding the correlation between intelligence and achievement an important validity issue. Some (e.g., Ackerman 2022) argue that school grades should be used to examine the relationship between intelligence and achievement. Others (e.g., Jensen 1998) noted that grades are “more influenced by the teacher’s idiosyncratic perceptions of the child’s apparent effort” (p. 278). We will present evidence of the relationship between intelligence and standardized achievement tests because these tests have demonstrated reliability. There is, however, a methodological limitation to this kind of research.

Studying the relationship between intelligence test score and achievement is complicated by the similarity in the items on traditional intelligence tests and achievement tests (e.g., vocabulary, arithmetic word problems) (Ackerman 2022). The similarity in content gives some intelligence tests an advantage over those such as the CAS2, which does not include verbal and quantitative test items (Naglieri and Bornstein 2003). The first large-scale study of the relationship between PASS scores and achievement was reported by Naglieri and Rojahn (2004). They examined the relationships between the PASS scores from the CAS and achievement scores from the Woodcock–Johnson Tests of Achievement–Revised (WJ-R; Woodcock and Johnson 1989) for a nationally representative sample of 1559 students and found an average correlation of 0.70. 

Naglieri and Otero (2017) reported the correlations between several intelligence tests and achievement tests using two methods. First, the average correlation among all the scales on each intelligence test with an achievement test was computed. Second, the average of the scales on the intelligence tests that clearly did not demand knowledge were obtained. This enabled an understanding of how each intelligence test was correlated with achievement when the most achievement-like scale on the intelligence test was excluded. This procedure was conducted for the WISC-V and WIAT-III using data from the WISC-V manual (Wechsler 2014), Woodcock–Johnson IV (McGrew et al. 2014), and the K-ABC-II (Kaufman and Kaufman 2004). The findings showed that the correlation between each of these tests and achievement was higher when the scales that demand verbal knowledge were included. For example, the best explanation for why the Wechsler Verbal Comprehension scale and the WIAT-III were so highly correlated is the similarity in content across the two tests. Some (e.g., Lohman and Hagen 2001) argue that this is evidence of validity. However, others may suggest that correlations between achievement and intelligence tests that contain questions that demand, for example, knowledge of words and arithmetic may be inflated because of the shared content. The correlations between intelligence tests that do not require knowledge and achievement tests may provide a more accurate estimate of the relationship between cognitive ability and achievement. What was most important was the correlation between the CAS and achievement; it was the highest of any of the correlations obtained with tests that demanded knowledge. A recent meta-analysis of the relationship between PASS scores on the CAS and achievement revealed the same findings.

Georgiou et al. (2020) examined the relationships between PASS scores from the CAS with reading and math in 93 independent samples. They found that (a) PASS “cognitive processes (operationalized with CAS) can produce correlations that are stronger than those derived from popular IQ batteries (e.g., WISC) that include tasks (e.g., Arithmetic, Vocabulary) whose content is often confounded by school learning;” (p. 10) (b) PASS “processes have direct implications for instruction and intervention programming. For example, cognitive strategy instruction based on PASS processes has been found to improve children’s math calculation (Iseman and Naglieri 2011) and PASS Reading Enhancement Program (PREP) has been found to improve children’s decoding (Papadopoulos et al. 2004) and reading comprehension” (Mahapatra et al. 2010) and (c) “the present meta-analysis adds to a growing body of research examining the role of intelligence in academic achievement (e.g., Peng et al. 2019; Roth et al. 2015) suggesting that there are significant benefits if we conceptualize intelligence as a constellation of cognitive processes that are linked to the functional organization of the brain” (p. 10).

### 4.2. Intelligence Test Profiles

There has been and continues to be considerable controversy about which scores on the various intelligence tests should and should not be interpreted when practitioners examine a profile of scores. The issue is centered around the amount of support that has been found for subtest, scale, or full-scale level interpretation. For example, Kaufman advocated for interpretation at many levels (Kaufman et al. 2016). Other researchers argue that valid interpretation of the many scores typically provided “is dependent on how precisely each score reflects its intended construct and whether it provides unique information independent of other constructs” (Watkins and Canivez 2022, p. 619). These researchers have found that the most valid score on, for example, the Wechsler Intelligence Scale for Children Fifth Edition (Canivez et al. 2017; Watkins and Canivez 2022), Stanford–Binet Fifth Edition (Canivez 2008), Differential Abilities Scales (Canivez and McGill 2016), and the Woodcock–Johnson Fourth Edition (Dombrowski et al. 2017) is the total score that estimates general ability, or *g.* Moreover, the reanalysis of John Carroll’s (1993) survey of factor-analytic studies conducted by Benson et al. (2018) came to the same conclusion. They wrote that nearly all the specified abilities presented by Carroll “have little-to-no interpretive relevance above and beyond that of general intelligence” (p. 1028). These researchers have published many studies and have consistently found that practitioners should only report the total score, which represents general ability, and not the subtests or scales that are provided. There has been only one exception—the PASS scales of the CAS.

Canivez (2011) concluded that sufficient variance was attributed to the PASS scales on the Cognitive Assessment System, supporting their interpretation. The factorial structure of the CAS2 has also been examined. Papadopoulos et al. (2023) conducted a series of analyses using the standardization sample of the CAS2. Their study included an analysis of four cognitive factors (i.e., correlated model), a general g factor (i.e., one- and second-order factor models), or a combination of the two (i.e., bi-factor models). The results revealed that the correlated PASS model accounted for the inter-subtest covariation of the PASS neurocognitive abilities better than the unitary *g* factor or the bifactor models. Furthermore, factorial invariance analysis provided evidence that the PASS model, as a measure of cognitive processing or intelligence, was the same between genders. The factor analytic research provides important information about the structure of intelligence tests and gives direction to practitioners about which scores to interpret, but it is equally important to examine intelligence test profiles across disabilities.

**Figure 2:**
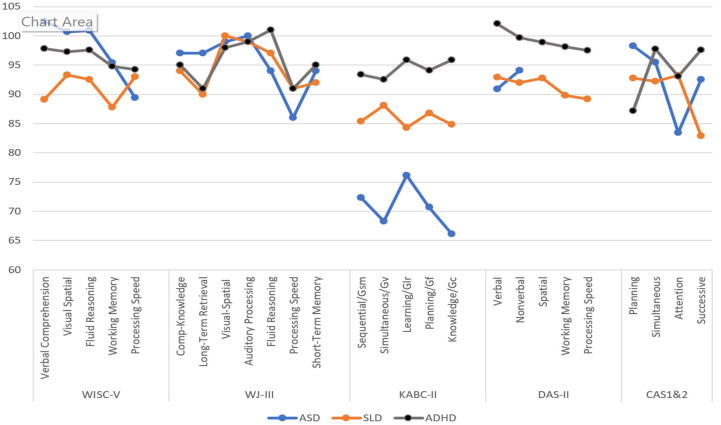
Scale profiles on various intelligence tests for samples with ASD, SLD, and ADHD. Note: DAS-II scores for individuals with autism were only available for the Verbal and Nonverbal scales.

Otero and Naglieri (2023) addressed the utility of scale variability by examining profiles for individuals with ADHD, SLD, and ASD. Rather than an examination of subtest scores, they reported the scores on the scales provided in each test. They chose this approach because scales have higher reliability than subtests, and scales typically correspond to some intellectual construct identified by the authors. This level may also provide information that could be used to identify a specific pattern of strengths and weaknesses relevant to a student’s learning difficulty and may have diagnostic value. The data provided in Figure 2, largely obtained from the respective tests’ technical manuals, must be considered with recognition that the samples were not matched on demographic variables across the various studies, the accuracy of the diagnoses may not have been verified, and some of the sample sizes were small. Notwithstanding these limitations, the findings provide insights into the extent to which these tests are likely to yield profiles that could offer insight into the groups’ cognitive variability.

The profiles for students with SLD in reading decoding (dyslexia) across the WISC-V, KABC-II, and DAS-II scales show little variability (4–6 points). The WJ-III scores were all within the average range (90+), with a range of 10 points between the Visual-Spatial and Long-Term Retrieval scales. (More recent data for the WJ-IV is not provided in their Manual). The PASS scores also varied by 10 points, but the lowest score of 83 was on the Successive processing scale, and the other three scales were in the average range. The patterns for students with ADHD were also provided.

There was a small variability of scores for the ADHD samples on the WISC-V, KABC-II, and DAS-II (3–5 points). Although the WJ-III scores varied by 10 points, all the scales’ scores were within the average range. The PASS scores varied by 11 points; the highest score was on the Successive and Simultaneous scales (98), and the lowest score was on the Planning Scale (87). The results for the CAS included the CAS2, as well as values reported by Naglieri et al. (2003, 2004) and Van Luit et al. (2005). 

The results for students with ASD showed a small difference between the Verbal and Nonverbal scales’ scores on the DAS-II. The KABC-II scores varied by 10 points, with all scores between 66 and 76. The WISC-V and WJ-III scores varied by 13 and 14 points, respectively, but nearly all the scores were in the average to low average ranges, with Processing Speed the lowest. The PASS scales showed the most variation, from a high of 98 on Planning to a low of 83 on Attention. The examination of these profiles provides a preliminary picture of the extent to which samples with different diagnoses are associated with different intelligence test results.

Huang et al. (2010) examined PASS scores from the CAS standardization sample, referred to as the general education group (N = 1692), and a collection of students identified as having a learning disability (N = 367) from research by Brams (1999) and Johnson (2001). They used a cluster analysis methodology to identify unique groups based on their PASS scores. Ten distinct groups were found for the general education sample, and 12 different groups were identified for the sample with learning disabilities, as shown in Table 3 and Table 4, respectively. The profiles that were found provide some indication of the relationship between PASS score variability and different diagnostic groups.

The 10 groups of students identified in the general education sample vary from those with consistently high PASS scores (clusters 1 and 2) to those with all low PASS scores (cluster 10). These two clusters have PASS scores that could have implications for instruction and eligibility determination. For example, cluster 1 in the general education (GE) sample would likely include students with scores high enough to qualify for a gifted education program. There is also the possibility that students within this cluster with overall high scores might also show significant variability in PASS scores that have instructional implications and may even suggest a learning disability, as shown by Georgiou et al. (2020). They found that 54% of their sample had a PASS score that was significantly lower than each student’s average PASS score; 8% had a PASS score that was low in relation to the student’s average and less than 90 (which suggests a disorder in a basic psychological process); and 4% had both a PASS disorder and similarly low academic score, which could support the presence of a specific learning disability. Clusters 2–5 in the GE sample show variability of 14–21 points, with the smallest range found being cluster 10. This group’s scores ranged from 79 to 81 and suggests a sample that likely includes students with intellectual disabilities.

Huang concluded that the 10 profiles in the general education sample suggest that there were groups of students with different PASS patterns reflecting different learning strengths and weaknesses, which could have implications for instruction. Similarly, the 12 profiles for the sample of students with different kinds of learning disorders support the idea of associating PASS scores with different learning disabilities. They stated that: “the presence of various patterns of PASS cognitive processes provides initial, yet promising evidence that interpretation at the composite level using the CAS is useful for the cognitive assessment approach for identifying LD in children” (p. 27).

### 4.3. Diagnostic Implications

An essential step in understanding if a neurocognitive processing strength corresponds to an academic strength and a neurocognitive processing weakness corresponds to an academic weakness is achieved by comparing PASS and achievement test scores. Comparisons between ability (PASS neurocognitive) and achievement (reading, math, etc.) can be efficiently accomplished using the CAS2 because the PASS test items do not rely heavily on knowledge. That is, there are no vocabulary, general information, or arithmetic questions on the CAS2 (see Naglieri and Otero 2018 for more discussion), which makes the analysis of the pattern of strengths and weaknesses across intelligence and achievement measures free from content overlap. It may be useful for practitioners to use the PASS scores when considering the identification of a specific learning disability described in the IDEA as a disorder in one or more of the basic psychological processes that are associated with academic failure. 

There are several methods for detecting a pattern of strengths and weaknesses (PSW) that could be used as part of the process of identifying a student with, for example, a specific learning disability (SLD). Naglieri (1999), Hale and Fiorello (2004), and Flanagan et al. (2007) put forth a method for finding a combination of differences as well as similarities in scores across academic and cognitive tests to establish the presence of a disorder in one or more cognitive processes and its correspondence to deficits in academic skills. The approach used to operationalize a PSW using PASS scores from the CAS2 is called the Discrepancy Consistency Method (DCM). The method involves an examination of the variability of PASS and academic achievement test scores, which has three parts: two discrepancies and one consistency that form a pattern of strengths and weaknesses. A PASS scale discrepancy is found if there is a significant difference among the four scales relative to the child’s overall performance, with one or two PASS scores that are substantially below what would be considered typical (the normal range). A second discrepancy could be found between the PASS strengths and academic weaknesses. The consistency portion of the DCM is found when achievement scores are consistent with the low PASS scores. Such a finding suggests that a child may have a disorder in the basic psychological processes necessary for SLD identification (Naglieri 2005, 2011; Naglieri and Otero 2017; Naglieri and Feifer 2018). 

**Figure 3:**
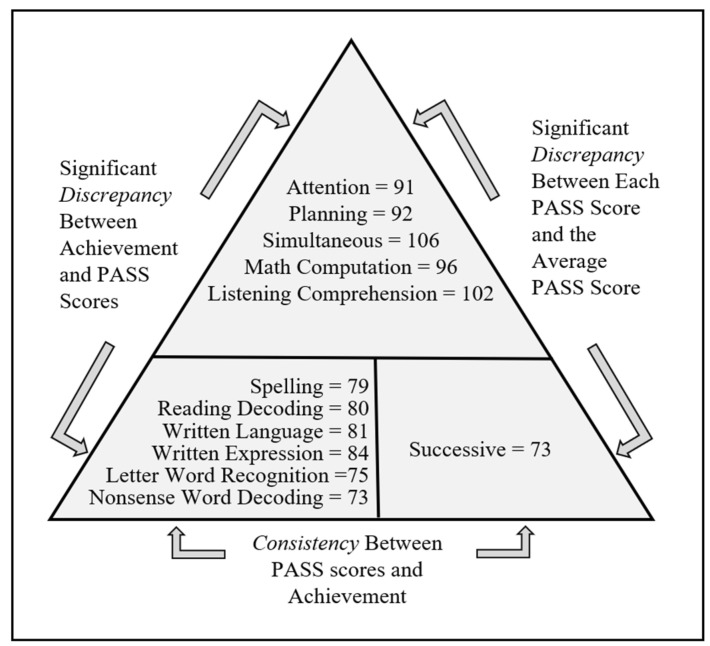
Example of the Discrepancy Consistency Method for communicating findings across PASS and achievement test scores.

Figure 3 provides an illustration of the Discrepancy Consistency Method. In this example, the PASS and achievement test scores fall into three groups. First, we notice the student has strengths in Simultaneous processing with average scores in Attention and Planning. The Successive processing score of 73 is significantly lower than the average of the four PASS scores. It is important to note that the top of the triangle provides strengths in cognition and achievement, which may have relevance to intervention design. There is evidence of weaknesses in academic skills, ranging from a score of 73 on reading nonsense words to a score of 84 in written expression. These weaknesses are consistent with the Successive processing score of 73, according to the values required for significance provided by Naglieri and Otero (2017) using the PASS Score Analyzers for comparing PASS and achievement test scores (Naglieri 2020).

### 4.4. Intervention

One of the most important tasks associated with a comprehensive assessment is explaining how a student learns best, what obstacles to learning may exist, and how this information may inform instruction. Intellectual abilities that can be easily explained to teachers, parents, and, most importantly, the students can make this task more informative. The PASS theory provides the practitioner with ways to explain how a person learns best (i.e., PASS strength), what obstacles to learning may exist (i.e., PASS weakness), and what can be done to maximize learning (Naglieri and Feifer 2017). Interpretation of the PASS scales (not subtests) is based on the definitions of the constructs and the following descriptions that are easy to explain to a teacher, parent, and student: 

Planning is a kind of thinking used when you think about *how* to do something. 

Attention is used when you focus your thinking on something and resist distractions.

Simultaneous processing is used when you think about how ideas or things go together. 

Successive processing is used when you think about the sequence of actions or sounds. 

These PASS scores can form a profile of an individual student’s learning strengths and weaknesses that can help determine which kinds of instruction should be considered (Naglieri and Feifer 2017). Naglieri and Pickering (2010) provide resources for interventions that are aligned with the PASS theory, rendered in brief handouts for teachers, parents, and students. There are also other resources for applying the PASS theory to academic instruction and remediation—for example, the PASS Remedial Program (PREP; Das 2000) and Planning Facilitation (Naglieri and Pickering 2010).

PREP was developed as a remedial program based on the PASS theory of cognitive functioning (Das et al. 1994). The program is designed to encourage the use of Simultaneous and Successive processes that underlie reading for students aged 7–10 years. The program avoids the direct teaching of word-reading skills such as phoneme segmentation or blending because it is based on the premise that the transfer of learning is best facilitated through inductive rather than deductive inference (Das 2009). PREP is structured so that strategies used to solve nonacademic tasks are generalized to tasks that demand academic content. Students are provided the opportunity to develop strategies in their own way to use Simultaneous and Successive neurocognitive processes (Das et al. 1995) within the context of reading and spelling (Das et al. 1994). Several studies have demonstrated the efficacy of PREP for the enhancement of reading and reading comprehension (Boden and Kirby 1995; Carlson and Das 1997; Das et al. 1995; Parrila et al. 1999).

Another intervention approach based on PASS is Planning Facilitation, an instructional method first studied by Naglieri and Gottling (1995), which encourages students to be strategic (use Planning) when they complete reading and math tasks. The initial concept for planning facilitation was inspired by the work of Cormier et al. (1990) and Kar et al. (1992). Cormier et al. (1990) found that overt verbalization improved scores on a complex task and that the intervention was particularly effective in improving scores for children low in Planning. Kar et al. (1992) examined the degree to which students with poor or good Planning scores benefited differently from a verbalization intervention like the one used by Cormier et al. (1990). They found that students who had low Planning scores benefited more from the verbalizations of strategies than those with high Planning. These studies suggested that an intervention that encourages verbalizations about how to complete a task, the value of noting the important parts of a problem, and increased awareness of new ways to achieve the goal was differentially effective based on a student’s Planning score. These studies did not, however, involve academic tasks such as math or reading, a limitation addressed by Naglieri and Gottling (1995, 1997). Naglieri and Gottling (1995) provided one-on-one sessions to students with learning disabilities using the Planning Facilitation method and math taken from the school curriculum. Students were given ten minutes to complete math worksheets, followed by five minutes of self-reflection guided by a tutor, and then ten more minutes to complete another math worksheet. The tutor gave prompts such as, “What did you notice about how you did the work? and What could you have done to get more correct?” The results showed that the intervention helped all the students, especially those low in Planning. The second study by Naglieri and Gottling (1997) also included students with learning disabilities. The teachers facilitated group discussion in seven baseline sessions and 21 intervention sessions, during which questions were presented to help students reflect on how they completed the math worksheets. The teachers asked questions such as, “What could you have done to get more correct” and “What will you do next time?” The intervention designed to facilitate a planful approach to math given by teachers to their classes had differential effects depending upon the PASS profile. That is, students with low Planning scores improved more than those with high Planning scores because this instruction met their need to be more strategic when completing math computation problems. 

Naglieri and Johnson (2000) conducted a study to determine if the Planning Facilitation method given by regular classroom teachers would have differential effects depending on the PASS profiles of the students with learning disabilities and mild mental impairments. The students completed math worksheets during baseline and intervention phases, and PASS scores were obtained using the CAS. The findings confirmed previous research. Students with a cognitive weakness in Planning improved considerably (effect size of 1.4) compared to those with an Attention weakness (effect size of 0.3), Simultaneous weakness (effect size of −0.2), or Successive processing weakness (effect size of 0.4) and those without a weakness (effect size of 0.2). The authors concluded that the Planning Facilitation method, “which does not use teacher scripts or rigidly formatted procedures, can be replicated” (p. 595) and that the cognitive strategy instruction is especially helpful for the students who need it the most—those with low Planning scores. The next study on this method involved reading comprehension.

The purpose of a study by Haddad et al. (2003) was to determine if the Planning Facilitation method would have a different impact on reading comprehension for students with different PASS profiles from the CAS. The students’ pre- and post-reading comprehension scores were compared for those with a PASS weakness. The results showed that students with a weakness in Planning benefited from the Planning Facilitation method (effect size = 0.52). Students with no weakness and those with a Successive processing weakness (effect size = 0.06) did not benefit from the intervention. This study showed that helping students utilize Planning while completing a reading comprehension task had beneficial results, similar to the findings for math and nonacademic tasks. 

Iseman and Naglieri (2011) examined the effectiveness of the Planning Facilitation method for students with learning disabilities and ADHD randomly assigned to a control or experimental group. The students in the experimental group were given the Planning Facilitation method, and the control group received additional math instruction from the regular teacher. The results showed that students in the experimental group benefited (effect size = 0.85) from this instructional method, which encourages students to reflect on how they complete the work (i.e., use executive function). The comparison group who received math instruction from the regular teacher did not do as well (effect size 0.26). The intervention helped students in the experimental group develop and use more effective planning strategies when completing the math worksheets. In addition, students in the experimental group also showed significantly greater improvement on the Math Fluency subtest of the Woodcock–Johnson Achievement test and the WIATT-II Numerical Operations subtest. The authors concluded, “These results indicate not only did those students with ADHD benefit from planning strategy instruction in classroom math, as shown by their improvement on the worksheets, but also that they were able to transfer learned strategies to other measures of mathematics, suggesting far transfer of skills” (p. 191). In addition, the experimental group’s math scores were significantly greater than the control group one year later. 

The results from this study support the previous studies on this instructional method called Planning Facilitation. The method was designed to avoid the direct teaching of strategies because transfer of learning is best achieved through inductive rather than deductive inference, as described in the section above about PREP (Das 2009). The study by Iseman and Naglieri (2011) is especially important because it used a randomized design and showed transfer from classroom math to norm-referenced tests of math achievement. In addition, the improvement found for students with ADHD is particularly important because researchers have found small effect size improvement in academic skills for students with ADHD (DuPaul et al. 2012; Reid and Maag 1998). Collectively, these intervention studies illustrate a relationship between PASS test scores and classroom instruction, as well as suggest a connection between intervention effectiveness and PASS profiles. 

## 5. Conclusions

We have provided a short historical perspective on the state of intelligence testing in the 2020s and emphasized that the tests most widely used since the early 1900s have two critical limitations. Traditional intelligence tests were not built on a theory of intelligence, and they include content that is indistinguishable from questions on achievement tests (Schneider 2013), which distorts the test scores for those with limited opportunity to learn. This appears to be a factor in the differences observed across race and ethnicity. The possible consequences of these limitations were anticipated by Bronner et al. (1927) when they wrote: “inaccuracy of psychological diagnosis [may result] in positive harm to the individual and hinders the development of scientific psychology (p. v)”. This caution foretold the American Psychological Association’s Apology to People of Color for APA’s Role in Promoting, Perpetuating, and Failing to Challenge Racism, Racial Discrimination, and Human Hierarchy in the U.S. (APA 2021). We have presented summaries of research that suggest that a theory of intelligence that focuses on basic psychological processes defined by brain function and explicitly developed to minimize formal knowledge may offer the potential for greater validity and equity and thereby provide a possible remedy to address APA’s Apology. 

Change in any field is not always easy. We hope that the information summarized here provides some evidence to support a consideration of a significant change. It is also important to recognize that Standards for the practice of psychology inform us of our professional obligations, which, according to the American Psychological Association, “are intended to facilitate the continued systematic development of the profession and to help facilitate a high level of practice by psychologists.” (https://www.apa.org/practice/guidelines/child-protection, accessed on 3 July 2024). Kelly (2023) described the National Association of School Psychologists (NASP) Ethical Standards related to the practice of intellectual assessment, especially as it relates to equitable assessment. He noted that the NASP standards state that school psychologists should promote fairness and social justice (Guiding Principle 1.3), that they work as change agents to correct school practices that are unjustly discriminatory, and they do not engage in or condone actions or policies that discriminate (Standard 3.2). It is, therefore, important for all professionals who use cognitive measures to carefully examine all aspects of the validity of intelligence tests, especially as it relates to fairness, when making test selection decisions. 

It is easy to rely on tests that are popular and already familiar to us. However, as we have shown, after a century of use, intelligence tests built without a firm basis in the theory of intelligence to guide test content have limitations. We suggest that researchers and practitioners recognize that an evolutionary step in the field of intelligence testing is needed, considering all we have learned in the past 100 years. The research presented here suggests that the PASS theory may provide a viable alternative to traditional intelligence tests. “To change our legacy [especially] with regard to systematic racism, we need to further heed the call and strongly pursue with the utmost urgency [new] streams of research and quickly leverage the findings to put into practice the mechanisms needed to drive real change (Goldstein et al. 2023, p. 12)”. 

## Figures and Tables

**Table 1:** PASS, functional units, and Neuro-networks.

PASS Processes	Functional Units	Neuro-Network
Planning	3rd Functional Unit	This neurocognitive process provides for the programming, regulation, and verification of behavior and is responsible for behaviors such as asking questions, solving problems, self-monitoring, regulation of voluntary activity, conscious impulse control, various linguistic skills such as spontaneous conversation, and the complex expression of personality. Planning is associated with the prefrontal lobes of the brain and interacts with the first and second units and their associated networks.
Attention	1st Functional Unit	This neurocognitive process provides the brain with the appropriate level of arousal or cortical tone, as well as directive and selective attention. The first functional unit, along with its related networks, allows for orientating, sustaining, and reorienting attention to what has relevance at any moment in time. Attention (i.e., cortical arousal) is associated with the brain stem and reticular activating system, which interacts with the default mode network and activation of the ventral and dorsal attention networks. It is also associated with the fronto-parietal system, which facilitates Simultaneous and Successive processes.
Simultaneous and Successive	2nd Functional Unit	Simultaneous neurocognitive process provides for the understanding and use of the interrelated nature of information. Successive neurocognitive processing provides for the understanding and use of sequential information. Activation of the frontal, parietal, and temporal regions is key to both Simultaneous and Successive processing. This region is considered the association cortex, which has many interrelated functions (such as attention, spatial representation, working memory, eye movements, an assortment of other sensory information, and the guidance of actions).

**Table 2:** Standard score differences by race and ethnicity across intelligence tests.

		Race	Ethnicity
Tests that require knowledge		Mn = 9.4	Mn = 6.6
	Otis–Lennon School Ability Test	13.6	-
	Stanford–Binet IV	12.6	-
	WISC-V	11.6	-
	WJ-III (normative sample)	10.9	10.7
	CogAT 7 Nonverbal	11.8	7.6
	CogAT 7-Verbal	6.6	5.3
	CogAT 7-Quantitative	5.6	3.6
	CogAT-Nonverbal	6.4	2.9
	CogAT-Total (V, Q & NV)	7.0	4.5
	K-ABC II Fluid-Crystallized Index	9.4	9.8
	K-ABC II Mental Processing Index	8.1	8.2
	WISC-V (statistical controls)	8.7	-
Tests that require minimal knowledge		Mn = 4.3	Mn = 2.9
	K-ABC (normative sample)	7.0	-
	K-ABC (matched samples)	6.1	-
	KABC-II (adjusted for gender & SES)	6.7	5.4
	CAS-2 (normative sample)	6.3	4.5
	CAS (statistical control normative data)	4.8	4.8
	CAS-2 (statistical control normative data)	4.3	1.8
	CAS-2 Brief (normative samples)	2.0	2.8
	NNAT (matched samples)	4.2	2.8
	Naglieri General Ability Test-Verbal	2.2	1.6
	Naglieri General Ability Test-Nonverbal	1.0	1.1
	Naglieri General Ability Test-Quantitative	3.2	1.3

Note. These results were reported for the Otis–Lennon School Ability Test by Avant and O’Neal (1986); Stanford–Binet IV by Wasserman and Becker (2000); Woodcock–Johnson III race differences by Edwards and Oakland (2006) and ethnic differences by Sotelo-Dynega et al. (2013); CogAT7 by Carman et al. (2018) and Lohman (2012); WISC-V by Kaufman et al. (2016); K-ABC by Naglieri (1986); KABC:2 by Lichtenberger et al. (2006); Scheiber and Kaufman (2015); CAS by Naglieri et al. (2005); CAS-2 and CAS2: Brief by Naglieri et al. (2014b, 2014c); Naglieri Nonverbal Ability Test by Naglieri and Ronning (2000); and Naglieri General Ability Tests: Verbal, Nonverbal and Quantitative by Naglieri et al. (2022).

**Table 3:** PASS profiles for the general education sample.

	1	2	3	4	5	6	7	8	9	10
Planning	120	116	105	103	100	111	102	**87**	93	**79**
Simultaneous	118	103	114	99	*114*	102	**86**	101	92	***82***
Attention	119	*121*	96	107	106	106	99	**87**	*96*	**81**
Successive	115	102	*117*	*113*	100	**89**	99	*103*	**82**	**81**
Average PASS	118	110	108	106	105	102	96	94	91	81
Range	5	**19**	**21**	**14**	**14**	**23**	**15**	**16**	**14**	3

Note: PASS scores less than 90 are in bold font. Range of PASS scores within each group greater than 10 are in bold.

**Table 4:** PASS profiles for the learning-disabled sample.

	1	2	3	4	5	6	7	8	9	10	11	12
Planning	99	112	101	99	95	**86**	**87**	**82**	**85**	**88**	**78**	**76**
Simultaneous	115	106	100	105	95	103	97	**84**	96	**83**	**76**	**81**
Attention	99	117	103	102	95	97	**80**	**73**	**81**	91	**76**	**71**
Successive	118	98	102	**90**	100	**85**	**85**	98	97	**75**	**90**	**79**
Average PASS	108	108	102	99	96	93	87	84	90	84	80	77
Range	**19**	**19**	3	**15**	6	**18**	**17**	**25**	**15**	**16**	**14**	10

Note: PASS scores less than 90 are in bold font. Range of PASS scores within each group greater than 10 are in bold.

## Data Availability

No new data were created or analyzed in this study. Data sharing is not applicable to this article.
